# Coordinate Modulation of Glycolytic Enzymes and OXPHOS by Imatinib in BCR-ABL Driven Chronic Myelogenous Leukemia Cells

**DOI:** 10.3390/ijms20133134

**Published:** 2019-06-27

**Authors:** Viviana De Rosa, Marcello Monti, Cristina Terlizzi, Rosa Fonti, Silvana Del Vecchio, Francesca Iommelli

**Affiliations:** 1 Institute of Biostructures and Bioimaging, National Research Council, 80145 Naples, Italy; 2 Department of Advanced Biomedical Sciences, University of Naples “Federico II”, 80131 Naples, Italy

**Keywords:** BCR-ABL, OXPHOS, aerobic glycolysis, chronic myeloid leukemia

## Abstract

Since many oncogenes, including *BCR-ABL*, may promote the acquisition and maintenance of the glycolytic phenotype, we tested whether treatment of BCR-ABL-driven human leukemia cells with imatinib, a selective BCR-ABL inhibitor, can modulate the expression of key glycolytic enzymes and mitochondrial complex subunits thus causing alterations of glucose metabolism. BCR-ABL-driven K562 and KCL-22 cells were incubated with increasing concentrations of imatinib to preliminarily test drug sensitivity. Then untreated and treated cells were analyzed for levels of BCR-ABL signaling mediators and key proteins of glycolytic cascade and oxidative phosphorylation. Effective inhibition of BCR-ABL caused a concomitant reduction of p-ERK1/2, p-AKT, phosphorylated form of STAT3 (at Tyr705 and Ser727), c-Myc and cyclin D1 along with an increase of cleaved PARP and caspase 3 at 48 h after treatment. Furthermore, a strong reduction of the hexokinase II (HKII), phosphorylated form of PKM2 (at Tyr105 and Ser37) and lactate dehydrogenase A (LDH-A) was observed in response to imatinib along with a strong upregulation of mitochondrial complexes (OXPHOS). According to these findings, a significant reduction of glucose consumption and lactate secretion along with an increase of intracellular ATP levels was observed in response to imatinib. Our findings indicate that imatinib treatment of BCR-ABL-driven human leukemia cells reactivates mitochondrial oxidative phosphorylation thus allowing potential co-targeting of BCR-ABL and OXPHOS.

## 1. Introduction

Chronic myelogenous leukemia (CML) is a myeloproliferative disorder consistently associated with a t(9;22)(q34;q11) translocation which gives rise to the Philadelphia chromosome [1,2]. This translocation generates the *BCR-ABL* fusion gene that encodes for the chimeric BCR-ABL protein and drives neoplastic transformation of hematopoietic stem cells [2,3]. When fused with BCR, ABL tyrosine kinase is constitutively activated and retained within the cytoplasm where it can interact with several adaptor molecules creating multiprotein signaling complexes. BCR-ABL is reported to interact with Ras-mitogen-activated protein kinase (MAPK) pathway leading to increased proliferation, the Janus-activated kinase (JAK/STAT) pathway leading to impaired transcriptional activity, and the phosphoinositide 3-kinase (PI3K/AKT) pathway resulting in enhanced survival [4].

Imatinib mesylate is a small-molecule inhibitor of BCR-ABL tyrosine kinase activity that, by competing with ATP for the binding to the kinase domain of ABL, prevents chimeric protein autophosphorylation and downstream signaling leading to growth arrest and apoptosis [5]. Imatinib was approved for treatment of patients with chronic-phase CML and rapidly became the standard first-line therapy for those patients [6,7]. Although the mechanism of action of imatinib and its clinical efficacy have been well established, little is known about mechanisms underlying changes in energy metabolism of BCR-ABL driven CML cells in response to imatinib. Previous studies, using magnetic resonance spectroscopy, showed that imatinib treatment of BCR-ABL positive cells causes a decrease of glucose uptake and lactate production whereas it increases the production of intermediates of the Krebs cycle [8,9]. 

The aim of the present study was to test whether inhibition of BCR-ABL signaling by imatinib can modulate the expression of key glycolytic enzymes and mitochondrial complex subunits thus causing alterations of glucose metabolism.

## 2. Results

**Figure 1:**
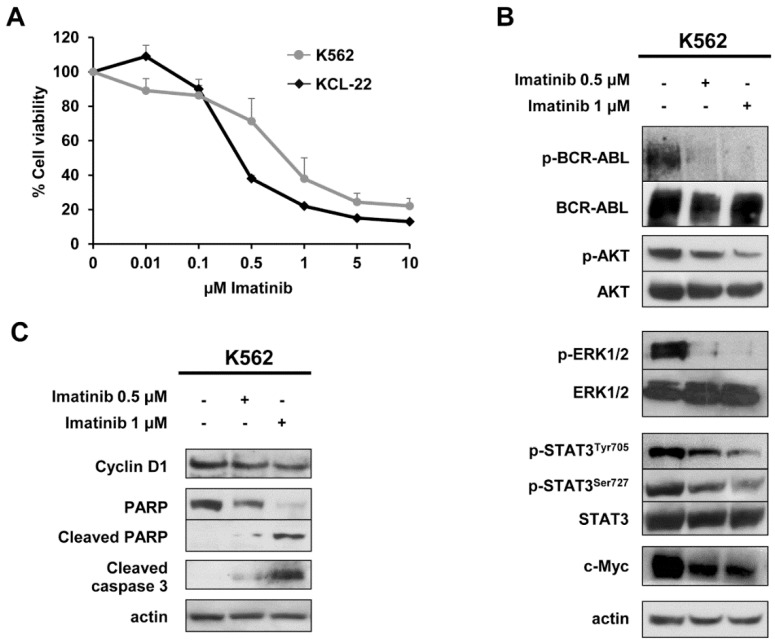
Modulation of BCR-ABL signalling by imatinib in chronic myelogenous leukemia (CML) cells. (**A**) Cell toxicity assays performed after 72 h of treatment with increasing concentration of imatinib showing an EC50 of 0.7 and 0.3 µM in K562 and KCL-22 cells respectively. Three independent assays were performed and data are expressed as mean ± SD. (**B**,**C**) Representative western blot analyses of whole-cell lysates obtained from K562 cells exposed to 0.5 and 1 μM imatinib or vehicle for 48 h. Actin served to ensure equal loading. At least three independent assays were performed.

The sensitivity of K562 and KCL-22 cells to increasing concentrations of imatinib was tested by MTS assay and the results are shown in Figure 1A. Viability of K562 and KCL-22 cells decreased in a dose-dependent manner showing an EC50 of approximately 0.7 and 0.3 µM, respectively, indicating sensitivity to imatinib. Furthermore, exposure of K562 cells to imatinib for 48 h caused a dose-dependent decrease of p-BCR-ABL, p-AKT, p-ERK1/2, p-STAT3^Tyr705^, p-STAT3^Ser727^ and c-Myc levels (Figure 1B) that lead to a decrease of cyclin D1 and a concomitant enhancement of cleaved PARP and cleaved caspase 3 levels (Figure 1C) indicating a drug-induced growth arrest and apoptosis. Similarly, a dose-dependent decrease of p-BCR-ABL and cyclin D1 was also observed in KCL-22 cells (Figure S1).

**Figure 2:**
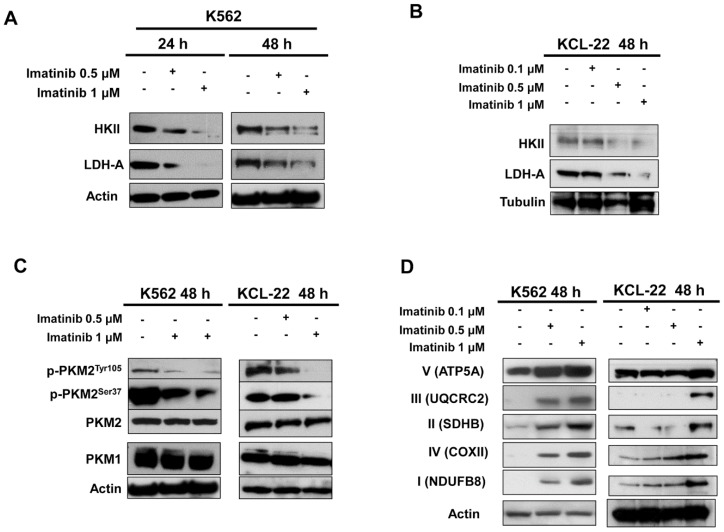
Protein levels of glycolytic enzymes and OXPHOS in CML cells. (**A**,**B**) Protein levels of HKII and LDH-A were assessed by western blot analysis of whole cell lysates from K562 (**A**) and KCL-22 (**B**) cells exposed to 0.1, 0.5, 1 μM imatinib or vehicle for 24 or 48 h. (**C**) Levels of p-PKM2^Tyr105^, p-PKM2^Ser37^ along with total form of PKM2 and PKM1 after 48 h treatment with imatinib assessed by western blotting. (**D**) Levels of OXPHOS in K562 and KCL-22 cells exposed to 0.1, 0.5 or 1 μM imatinib at 48 h. Actin served to ensure equal loading. At least three independent assays were performed.

Then levels of key glycolytic enzymes were determined in untreated and treated K562 and KCL-22 cells. A dose-dependent decrease of HKII and LDH-A expression (Figure 2A,B) was observed after 24 and 48 h treatment in K562 cells and confirmed in KCL-22 cells. Similarly, both cell lines treated for 48 h showed a dose-dependent decrease of p-PKM2^Tyr105^ and p-PKM2^Ser37^ levels (Figure 2C). In addition, 48 h imatinib treatment of K562 and KCL-22 cells caused a strong up-regulation of mitochondrial complex subunits (OXPHOS) indicating a concomitant reactivation of mitochondrial oxidative phosphorylation (Figure 2D). In contrast to OXPHOS increase, no significant changes of mitochondrial markers were found in response to imatinib as shown in Figure S2. 

**Figure 3:**
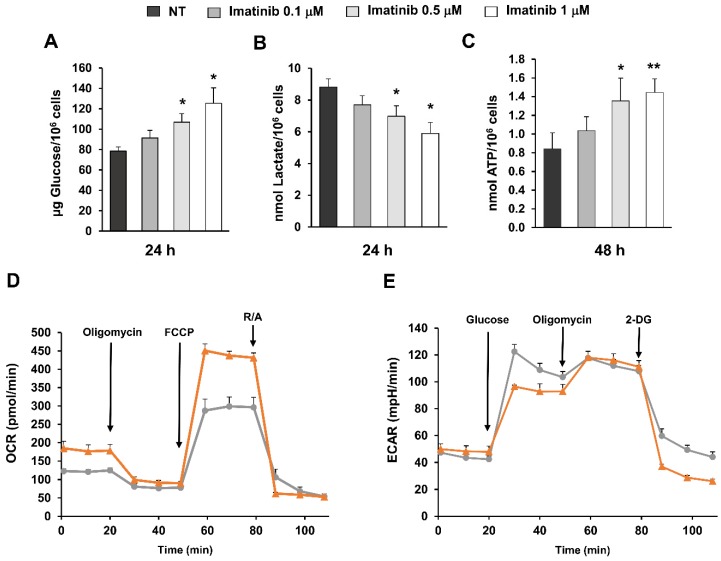
Functional assays, oxygen consumption rate (OCR) and extracellular acidification rate (ECAR) measurements in K562 cells exposed to imatinib. K562 cells were treated for 24 or 48 h with imatinib at the indicated concentrations. (**A**) Residual glucose levels were measured in conditioned media from untreated and treated cells. Data are expressed as μg of glucose normalized to 10^6^ cells. (**B**) Lactate levels were measured in conditioned media of untreated and treated cells and expressed as nmol of secreted lactate normalized to 10^6^ cells. (**C**) Intracellular ATP levels were determined in lysates of untreated and treated cells and expressed as nmol of ATP normalized to 10^6^ cells. The symbol * indicates significant differences versus untreated control with * *p* < 0.05 and ** *p* < 0.01. At least three independent assays were performed. (**D**,**E**) Simultaneous measurements of OCR (**D**) and ECAR (**E**) were performed in quintuplicate samples of untreated (grey line) and treated (orange line) K562 cells. All data are expressed as mean ± SE.

Then we evaluated glucose consumption, lactate secretion and ATP production in untreated and imatinib-treated K562 cells. A significant increase of glucose concentration was observed after 24 h in conditioned media of treated cells as compared to untreated controls (*p* < 0.05) indicating a lower glucose consumption (Figure 3A). A parallel significant decrease (*p* < 0.05) of lactate levels was found at 24 h in treated cells (Figure 3B) whereas intracellular ATP levels were significantly increased after 48 h of treatment with 1 µM imatinib (*p* < 0.01) (Figure 3C). In agreement with western blot analysis, functional assays indicate that imatinib treatment causes an early reduction of glucose consumption and lactate production through glycolysis followed by an increase of ATP indicating reactivation of oxidative phosphorylation. To confirm the results of functional assays, oxygen consumption rate (OCR) and extracellular acidification rate (ECAR) were measured in K562 cells exposed to 0.5 μM imatinib or vehicle for 48 h. An enhancement of both basal and maximal respiration rate was observed in treated K562 cells as compared to untreated controls (Figure 3D). Conversely, a reduction of ECAR was observed in treated cells as compared to untreated controls following the addition of glucose indicating a downregulation of glycolysis (Figure 3E). 

## 3. Discussion

The present study shows that imatinib treatment causes an early downregulation of the expression of glycolytic enzymes and an upregulation of mitochondrial complex subunits in BCR-ABL driven CML cells leading to a reduction of glucose consumption and an increase of intracellular ATP production. In particular, the decrease of HKII reduces the first step of glycolysis and glucose flux through the glycolytic cascade while the reduction of LDH-A caused a decrease of lactate secretion. Pyruvate kinase is a glycolytic enzyme that converts phosphoenolpyruvate to pyruvate [10,11]. Two isoforms of this enzyme, PKM1 and PKM2, are derived from alternative splicing of the primary RNA transcript of the PKM gene. PKM2 was found to be overexpressed in cancer cells and exists in a dimeric and tetrameric form associated with a low and high catalytic activity, respectively [12,13]. The reduction of p-PKM2^Tyr105^ levels in response to imatinib promotes tetramer formation and a high enzymatic catalytic activity leading to an increase of pyruvate that can be directed toward mitochondrial oxidative phosphorylation [14]. The enhanced pyruvate flux through Krebs cycle and the upregulation of OXPHOS may allow the increase of intracellular ATP levels. 

Phosphorylation of PKM2 at Ser37 was reported to induce its translocation into the nucleus where PKM2 serves as a transcriptional factor of several genes including c-Myc [15,16]. Imatinib causing a reduction of p-PKM2^Ser37^ may modulate the expression of a pool of c-Myc-dependent genes.

In our previous study, we showed the reversal of glycolytic phenotype in EGFR-driven non-small cell lung cancer cells and xenografts in response to EGFR inhibitors [17]. Here we demonstrated that similar results can be obtained by inhibiting BCR-ABL suggesting that different oncogene drivers can share common mechanisms to modulate energy metabolism. 

The upregulation of OXPHOS, the increase of intracellular ATP levels and the enhanced OCR in response to imatinib reflect the restoration of mitochondrial function. However, in these BCR-ABL driven cells, imatinib caused also an induction of apoptosis that leads to mitochondrial disruption. It is conceivable that in this context where glycolysis is downregulated and mitochondria are the only source of energy, the addition of OXPHOS inhibitors to imatinib would have a lethal effect on BCR-ABL driven cells. In this respect, the mitochondrial ATP-synthase inhibitor oligomycin A was reported to greatly sensitized leukaemia cells to imatinib predicting a potential benefit for patients treated with a combination of imatinib and OXPHOS inhibitors [18]. 

In conclusion, our study provided insights into the mechanisms underlying the changes of glucose metabolism in BCR-ABL driven cells treated with imatinib identifying OXPHOS as a potential target for therapy in combination with TKIs.

## 4. Materials and Methods

### 4.1. Cell Line and Treatment

The CML cell line, K562, was purchased from American Type Culture Collection whereas KCL-22 cells were kindly provided by Prof. B. Izzo and Dr. F. Quarantelli. K562 and KCL-22 cells were grown in IMDM and RPMI 1640 (Gibco, Thermo Fisher, Waltham, MA, USA) culture media, respectively, containing 10% fetal bovine serum (FBS), 100 IU/mL penicillin and 50 μg/mL streptomycin in a humidified incubator with 5% CO_2_ at 37 °C. Drug-induced toxicity was assessed by MTS assay (Promega, Madison, WI, USA) as previously described [19]. Briefly, cells were plated at a density of 5000/well in 96-well plates and then treated with increasing concentration (0.1–10 μM) of imatinib (Biaffin GmbH & Co KG, Germany) or vehicle for 72 h. The optical density (OD) was measured at 490 nm using microplate spectrophotometer, after 2 h incubation with MTS at 37 °C. At least three independent assays were performed and data are expressed as percentage of viable cells, considering the untreated control cells as 100%. EC50 values were calculated using GraphPad.

### 4.2. Immunoblotting Analysis

Whole cell lysates were prepared as previously described [20] after treatment with imatinib at 0.1, 0.5 or 1 μM for 24 or 48 h. Then untreated and treated cells were lysed with RIPA buffer (Sigma Aldrich, St. Louis, MO, USA), homogenized and centrifuged at 13,000× *g* at 4 °C for 20 min. Western blot analysis was performed using a standard procedure. 

Antibodies used for western blotting included mouse monoclonal antibodies recognizing BCR-ABL (1 µg/µl, Thermo scientific), STAT3 (1:1000), p42/44 MAP kinase (ERK1/2) (0.1 mg/mL) (Cell Signaling), HSP70 (2 μg/mL) (Santa Cruz Biotechnology), cytochrome C (1 μg/mL, BD Pharmingen, San Jose, CA, USA), α-tubulin (1 μg/mL), actin (1 μg/mL) (Sigma), PARP (1:1000, BD Pharmingen), the OXPHOS cocktail of 5 mAbs (Mitoscience, Eugene, OR; 1:1000) recognizes the following proteins: 20-kD subunit of Complex I (20 kD), COX II of Complex IV (22 kD), 30-kD Ip subunit of Complex II (30 kD), core 2 of Complex III (~50 kD), and F1α (ATP synthase) of Complex V (~60 kD); rabbit monoclonal antibodies against p-c-Abl^Tyr412^ (1:1000), Hexokinase II (1:1000), PKM2 (1:1000), LDH-A (1:1000), p-STAT3^Tyr705^ (1:1000) (Cell Signaling); rabbit polyclonal antibodies against PKM1 (1:1000, Abgent), p-AKT^Ser473^ (1:1000), PGC-1α (1:1000) (Santa Cruz Biotechnology), p-PKM2^Ser37^ (1:1000) (Signalway Antibody), p-PKM2^Tyr105^ (1:1000), phospho-p42/44 MAP kinase (ERK1/2) (1:1000), AKT (1:1000), cyclin D1 (1:1000), cleaved Caspase-3 (1:1000), c-Myc (1:1000), p-STAT3^Ser727^ (1:1000), (Cell Signaling). A commercially available ECL kit (GE Healthcare, UK) was used to reveal the reaction. At least three independent assays were performed.

### 4.3. Glucose Consumption, Lactate Secretion and Intracellular ATP

Cells were seeded in six-well flat-bottomed plates at a density of 3 × 10^5^ cells per well and then treated with imatinib at the indicated concentration for 24 or 48 h. Untreated and treated cells were analyzed for glucose and lactate levels in the conditioned media and for intracellular ATP levels. Briefly, after treatment, conditioned media were removed, centrifuged at 13,000× *g* at 4 °C for 10 min and then assayed for glucose and lactate concentrations using the Glucose Assay Kit (Sigma-Aldrich) and the Lactate Assay Kit (Sigma-Aldrich) following manufacturers’ instructions. Cells were simultaneously subjected to intracellular ATP determination using the ATPlite Luminescence Assay (Perkin Elmer; Waltham, MA, USA) following manufacturer’s instructions. Briefly, cells were lysed, incubated with the ATP reaction mixture for 5 min and then subjected to luminescence measurements. Absolute glucose, lactate and ATP levels were calculated from the corresponding standard curve and normalized to 10^6^ cells. At least three independent assays were performed.

### 4.4. Oxygen Consumption and Extracellular Acidification Rates

The oxygen consumption rate (OCR) and the extracellular acidification rate (ECAR) were determined using the Seahorse Extracellular Flux Analyzer (XF-96, Seahorse Bioscience, North Billerica, MA, USA). Briefly, K562 cells were plated at a density of 5 × 10^6^ cells in 10 cm petri dishes and then treated with 0.5 µM imatinib for 48 h. Then cells were plated on XF 96-well microplates and allowed to equilibrate. OCR was measured in basal conditions and after the subsequent addition of 5 μM oligomycin, 1.5 μM carbonylcyanide-4-(trifluoromethoxy)-phenylhydrazone (FCCP) and 1 μM rotenone/antimycin A. ECAR was simultaneously measured in basal conditions and after the subsequent addition of 10 mM glucose, 5 μM oligomycin and 100 mM 2-deoxyglucose. 

### 4.5. Statistical Analysis

Statistical analysis was performed using the software MedCalc for Windows, version 12.7.0.0, (MedCalc Software, Mariakerke, Belgium). Unpaired Student’s *t*-test was used to compare means. Differences between means were considered statistically significant for *p* < 0.05. 

## Supplementary Materials

Supplementary materials can be found at https://www.mdpi.com/1422-0067/20/13/3134/s1.
