# Supplementary material for: Coordinate Modulation of Glycolytic Enzymes and OXPHOS by Imatinib in BCR-ABL Driven Chronic Myelogenous Leukemia Cells
Source: Int J Mol Sci. 2019 Jun 27;20(13):3134. doi: 10.3390/ijms20133134 (PMC6651622; doi:10.3390/ijms20133134)
Supplement: Supplementary file 1 [file ijms-20-03134-s001.pdf]

Supplementary Figure

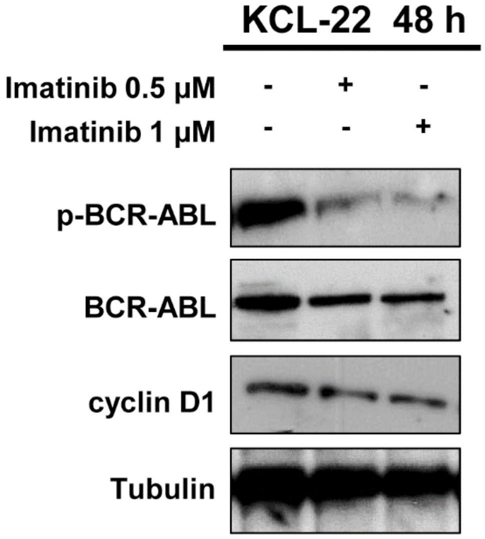

Figure S1. Levels of p-BCR-ABL, BCR-ABL and cyclin D1 in response to imatinib. Representative western blot analyses in whole cell lysates of KCL-22 cells exposed to imatinib for 48h

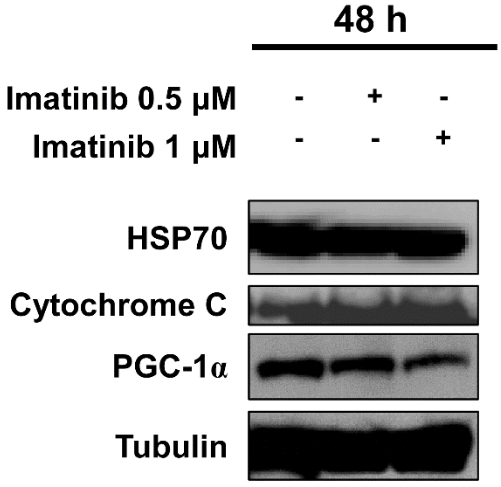

Figure S2. Levels of HSP70, cytochrome c and PGC-1α in response to imatinib. Representative western blot analyses in whole cell lysates of K562 cells exposed to imatinib for 48h
